# Construction and preservation of a stable and highly expressed recombinant *Helicobacter pylori* vacuolating cytotoxin A with apoptotic activity

**DOI:** 10.1186/s12866-021-02262-7

**Published:** 2021-08-18

**Authors:** Ling-Zhi Yuan, Xiao Shi, Dan Tang, Shao-Peng Zheng, Zhi-Ming Xiao, Fen Wang

**Affiliations:** 1 Department of Gastroenterology, the Third Xiangya Hospital, Central South University, Changsha, Hunan 410013 China; 2 Hunan Key Laboratory of Non-resolving Inflammation and Cancer, Central South University, Changsha, Hunan 410013 China

**Keywords:** *Helicobacter pylori*, VacA recombinant protein, Expression, Purification, Preservation, Apoptosis

## Abstract

**Background:**

*H. pylori* is closely related to the occurrence and development of various digestive gastritis, peptic ulcer and mucosa-associated lymphoid tissue (MALT) lymphoma. *H. pylori* is also a class I carcinogen of gastric cancer. *VacA* is the only exocrine toxin of *H. pylori*, which plays a very important role in the pathogenesis of *H. pylori*. The production of *VacA* in natural circumstances is complex with heavy workload and low yield. Therefore, it is very important to obtain recombinant VacA protein which is stable and biologically active. This study therefore aims to explore the expression, purification and stable storage of *VacA* toxin of *H. pylori* in E.coli, and to provide experimental basis for further exploration of the role of VacA in *H. pylori* -induced inflammation of cancer.

**Results:**

A 2502-bp fragment and *VacA* gene were identified. An 89.7-kDa VacA^34–854^ recombinant protein was expressed and purified from the recombinant engineering bacteria and was preserved stably in 50 mM acetic acid buffer (pH 2.9). The amount of the recombinant protein was larger in the inclusion bodies than in the supernatant. In addition, after a 24-h culture with VacA recombinant protein, GES-1 cells demonstrated evidence of apoptosis including early nuclear immobilization and clustering under inverted microscope and TEM. It was found that VacA recombinant protein induced apoptosis by TUNEL assay.

**Conclusions:**

A VacA recombinant protein that is stably and highly expressed and possesses pro-apoptotic activity is successfully constructed. The protein is stably preserved in 50 mM acetic acid buffer (pH 2.9).

**Supplementary Information:**

The online version contains supplementary material available at 10.1186/s12866-021-02262-7.

## Background

*Helicobacter pylori*, a main pathogen in the stomach, is closely associated with chronic gastritis, peptic ulcer disease, mucosa-associated lymphoid tissue lymphoma, and gastric cancer [1]. In 1994, the International Agency for Research on Cancer (IARC) officially listed *H. pylori* as a class I carcinogen for gastric cancer [2]. *H. pylori* infection is considered to be the most common infection worldwide with more than 50% of the world’s adult population being infected with the bacterium [3]. The prevalence of *H. pylori* is up to 90% in developing countries, while the annual recurrence rate is much higher than that in developed countries [4]. Persistent *H. pylori* infection, especially with a cytotoxic strain, leads to chronic gastric inflammation, tissue damage, increased cell proliferation and apoptosis, subsequent to precancerous lesions including atrophy, intestinal metaplasia, dysplasia, and finally gastric cancer [5]. In addition, Helicobacter pylori infection can induce a chronic immune response including persistent oxidative stress in the stomach, further leading to DNA damage that eventually can lead to gastric cancer [6, 7].

One of the most crucial toxins produced by *H. pylori* is the vacuolating cytotoxin A (*VacA*), which has been demonstrated to play a vital role in the pathogenesis of peptic ulcer disease and gastric cancer [8, 9]. *VacA*, a critical multifunctional virulence factor of *H. pylori*, responsible for eliciting several different effects on host cells including cellular vacuolation, cell death, depolarization of membrane potential, mitochondrial dysfunction, autophagy, activation of mitogen-activated protein kinases, inhibition of T cell activities, and some other immunomodulatory effects [10]. As a key toxin for *H. pylori* pathogenesis, *VacA* assists *H. pylori* to colonize the stomach by exerting multiple effects on epithelial cells in the human host [11]. Recent studies have revealed that *VacA* has turned out to be a potent immunomodulatory toxin. *VacA* has several mechanisms to help the bacteria evade immune response such as the disruption of phagosome maturation and the creation of fused phagosomes called megasomes, which prevent the destruction of the bacteria contained within [12]. Furthermore, *VacA* promotes immune tolerance and persistent *H. pylori* infection through its activities on T Cells and antigen-presenting cells [13]. These are all important virulence factors that *H. pylori* use to maintain a prolonged pro-inflammatory response while evading self-destruction. Moreover, considering the pivotal role of vacuolating cytotoxin A protein in *H. pylori* infection, *VacA* could be the best candidate for the construction of a recombinant vaccine [14].

Given the fascinating multi-functionality of *VacA* and its association with an augmented gastric cancer risk, extensive investigation into the structure and function of *VacA* is essential to fully understand the pathogenicity of *VacA* and to identify diagnostic and therapeutic strategies accordingly. However, the investigation has been hindered, to some extent, by the lack of a highly efficient method for the production of *VacA*. Several previous studies have reported that it is difficult to purify *VacA* from *H. pylori* and that the amount of protein that is produced is low [15]. Moreover, several recent studies have explored techniques through which to obtain a high-efficiency expression of VacA recombinant protein with biological-activity [16, 17]. However, these studies have weaknesses and limitations in that small fragments of VacA protein cannot fully reflect the function of the VacA protein and that these VacA recombinant protein preparations need to be acid-activated prior to use [18, 19]. Therefore, the aim of the present study was to establish a technique through which to construct and preserve a stable and highly expressed VacA recombinant protein that possesses the biological activity of promoting apoptosis.

## Results

### Construction of recombinant VacA-expressing plasmid

**Fig. 1:**
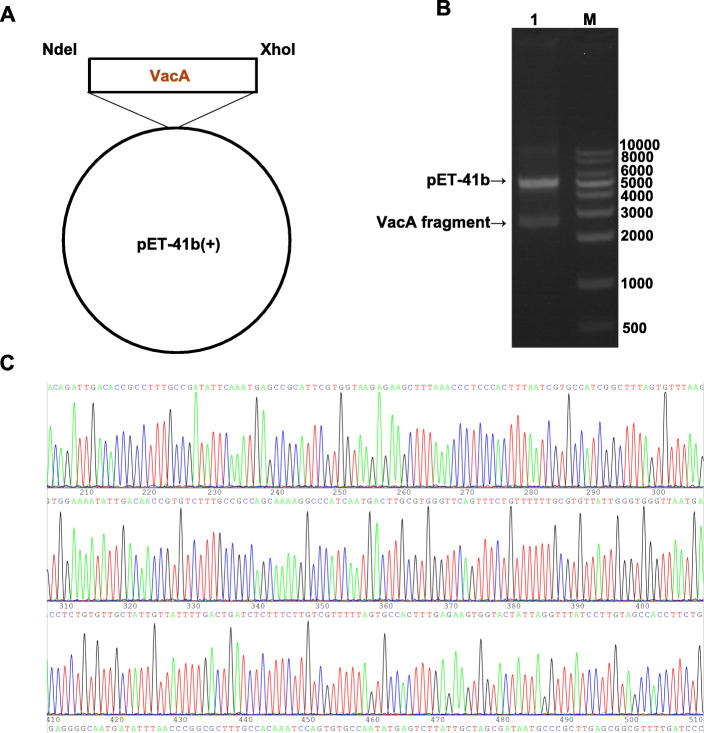
Successful construction of the recombinant plasmid. **A.** The construction diagram of a *vacA* carrier: *pET-41b-vacA*^*34–854*^. Gene name: *vacA*^*34–854*^; Gene length: 2502 bp; Carrier name: *pET-41b*; Enzyme cutting sites: *Nde*I + *Xho*I; C terminal integrates the 8*His label. **B.** *pET-41b*-*vacA*^*34–854*^ enzyme cutting identification. M: 1 Kb DNA ladder; Lane 1: recombinant plasmid *pET-41b*-*vacA*^*34–854*^ cut by *Nde*I & *Xho*I endonucleases. **C.** Positive clone sequencing of *pET41b-VacA*^*34–854*^ recombinant plasmid

A VacA-expressing plasmid was constructed successfully. Figure 1A shows that a single band, with a size of approximately 2502 bp, was generated from the recombinant plasmid. This finding is basically consistent with the expected fragment size. *Nde*I & *Xho*I endonuclease analysis of the recombinant plasmid revealed two corresponding electrophoretic bands, which were consistent with the sizes of the pET-41b plasmid linear vector (5932 bp) and the target *VacA* gene fragment (2502 bp; Fig. 1B). The sequencing of the recombinant plasmid revealed that the target gene fragment was completely consistent with the corresponding sequence of the *vacA* fragment in GenBank (Fig. 1C & Supplementary Fig. 1). This suggests that it was a specific target PCR product and that the recombinant plasmid was successfully constructed.

### Expression and purification of VacA recombinant protein

**Fig. 2:**
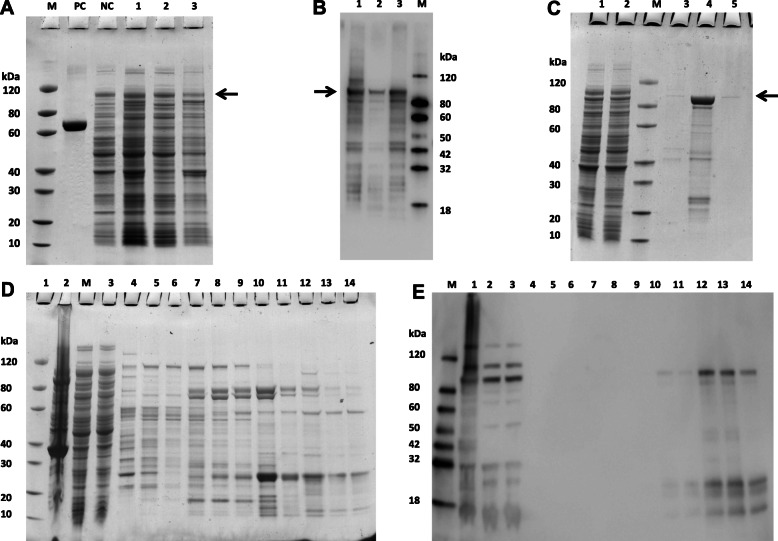
Expression and purification of the VacA recombinant protein in the pET-41b-vacA^34–854^- *E. coli* BL21(DE3) system. **A** and **B** The expression of the VacA recombinant protein in *E. coli* BL21(DE3) cells as detected by SDS-PAGE and western blotting, respectively. NC: no induced cell lysis; Lane 1: 15 °C in the induction of cell lysis of 16 h; and Lane 2: 15 °C in the induction of 16 h of cell lysis supernatant; Lane 3: 15 °C in precipitation of cell lysis induced 16 h. **C** VacA recombinant protein in the inclusion bodies purified with Ni column (IDA) as detected by SDS-PAGE analysis. Lane 1: Loading; Lane 2: Flow through; Lane 3: Elution with 20 mM imidazole; Lane 4: Elution with 500 mM imidazole; Lane 5: Ni resin after elution. Lane M: Protein marker. **D** and **E** VacA recombinant protein in the supernatant purified with Ni column (NTA) as detected by SDS-PAGE and western blotting, respectively. The column equilibration buffer consists of 50 mM Tris-HCl, 150 mM NaCl, pH 8.0, and wash buffer consists of 50 mM Tris-HCl, 1% Triton-X114, 8 M urea, 150 mM Nacl, PH 8.0). The target protein was eluted using a stepwise gradient of imidazole (i.e. 20, 50, 100 and 500 mM) and the results are shown as below: Lane 1: Pellet of cell lysate after centrifugation; Lane 2: Supernatant of cell lysate after centrifugation; Lane 3: Flow through; Lane 3–5: Elution with 20 mM imidazole; Lane 6–7: Elution with 50 mM imidazole; Lane 8–10: Elution with 100 mM imidazole; Lane 11–13: Elution with 500 mM imidazole; Lane 14: Ni resin after elution. Lane M: Protein marker. PC:positive control

IPTG at the concentration of 0.5 mM at 15 °C for 16 h efficiently induced the expression of the VacA recombinant protein in the *pET-41b-vacA*^*34–854*^-*E. coli* BL21(DE3) system (Figs. 2A & B). We attempted to further purify the target protein from the supernatant and inclusion bodies, respectively, and found that a large amount of protein was present in the pellet (Figs. 2C) and a small amount of protein was present in the supernatant (Figs. 2D & E).

### Refolding, preservation and identification of VacA recombinant protein

**Fig. 3:**
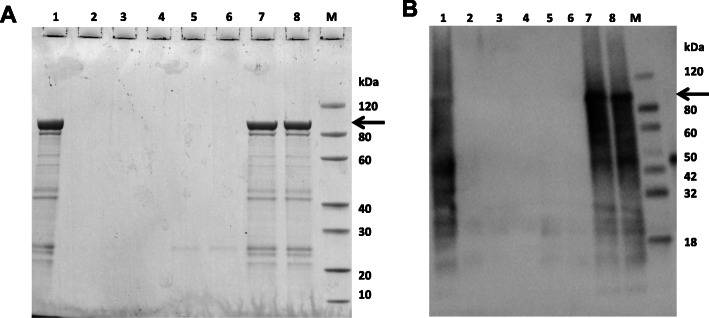
Renaturation of the target recombinant protein by various buffers as detected by SDS-PAGE (left) and western blotting (right). Lane M: Protein marker; Lane 1: Protein before refolding; Lane 2: buffer 1 (50 mM Tris,10% Gly, 150 mM NaCl, pH 8); Lane 3: buffer 2 (50 mM Tris,10% Gly, 150 mM NaCl, 0.1 mM DL-dithiothreitol (DTT), pH 8.0); Lane 4: buffer 3 (1× phosphate buffered saline (PBS), pH 7.4; Lane 5: buffer 4 (1× PBS, 10% GLy, 500 mM NaCl, pH 7.4); Lane 6: buffer 5 (1 × PBS,10% GLy, 500 mM NaCl, 0.1 mM DTT, pH 7.4); Lane 7: buffer 6 (20 mM Tris, 2 M urea, 400 mM Arg, 2.5 mM cysteamine, 0.25 mM cystamine, pH 8.5); Lane 8: buffer 7 (50 mM acetic acid, pH 2.9)

**Fig. 4:**
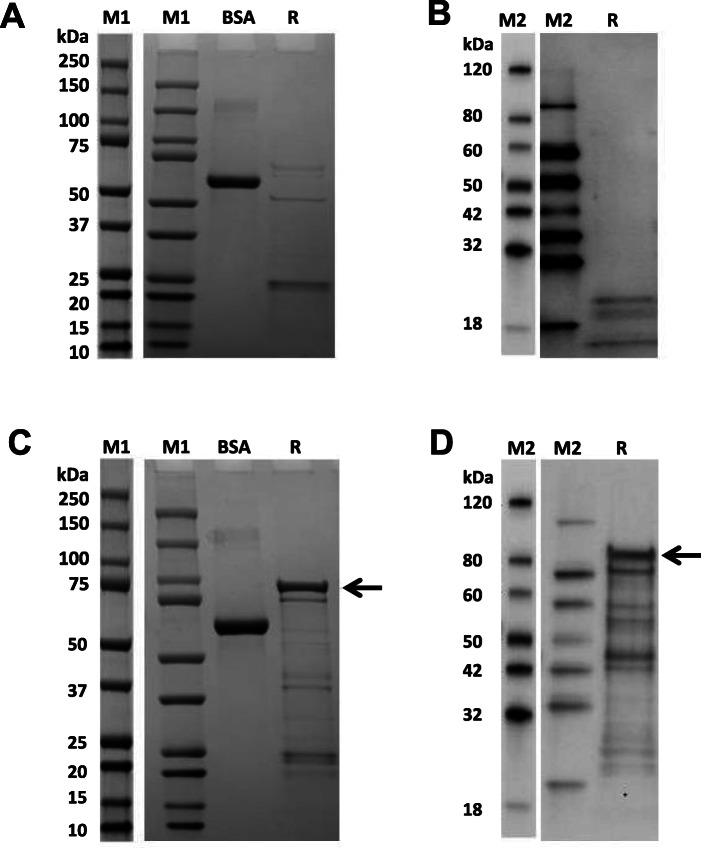
Expression of VacA recombinant protein in the supernatant and pellet as detected by sodium dodecyl sulfate polyacrylamide gel electrophoresis and western blotting. **A** and **B** Expression of VacA recombinant protein in the supernatant as detected by SDS-PAGE (left) and western blotting (right); **C** and **D** Expression of VacA recombinant protein in the pellet as detected by SDS-PAGE (left) and western blotting (right). BSA: bovine serum albumin(2.00 μg); R: VacA^34–854^ (2.00 μg); Both M1 and M2 are protein markers

A variety of protein refolding methods were attempted (Fig. 3). In the dialysis method, buffers 1–5 were all unsuccessful while the protein was recovered successfully in buffer 6 and buffer 7. However, Arg, which has an effect of promoting apoptosis, is present in buffer 6. The presence of Arg would, thus, interfere with the detection of the pro-apoptotic biological activity of the target VacA protein. Because of this, buffer 6 was not considered to be suitable as the final protein solution. Therefore, the target protein was successfully dissolved in buffer 7 (50 mM acetic acid, pH 2.9). Finally, an 89.7-kDa VacA recombinant protein molecular weight was obtained in the supernatant (Figs. 4A & B) and, in a larger amount, in the pellet (Figs. 4C & D).

### Apoptotic activity of VacA recombinant protein

**Fig. 5:**
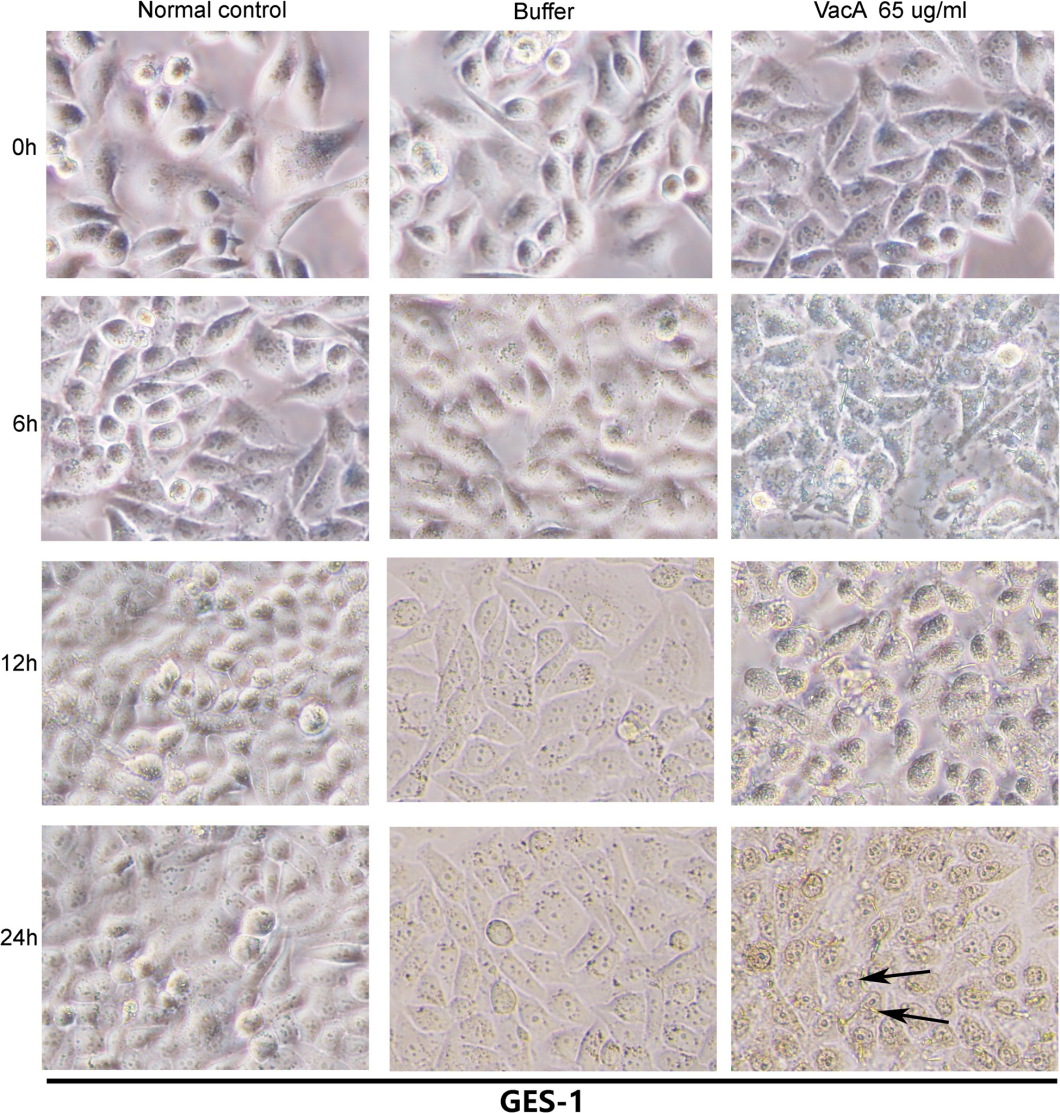
Morphological changes of GES-1 cells as observed by inverted microscopy after incubation with a high final concentration (65 μg/mL) of VacA recombinant protein for 24 h. Cellular morphological changes were visualized before incubation (0 h) and 6, 12 and 24 h after incubation by invert microscopy (Magnification × 100). After 24 h stimulation of VacA recombinant protein (as indicated by the arrow), significant cell morphological changes evident of apoptosis including early nuclear immobilization and clustering in GES-1 cells were observed. A large number of nuclei were retracted and chromatin in the nucleus converged to the edge

**Fig. 6:**
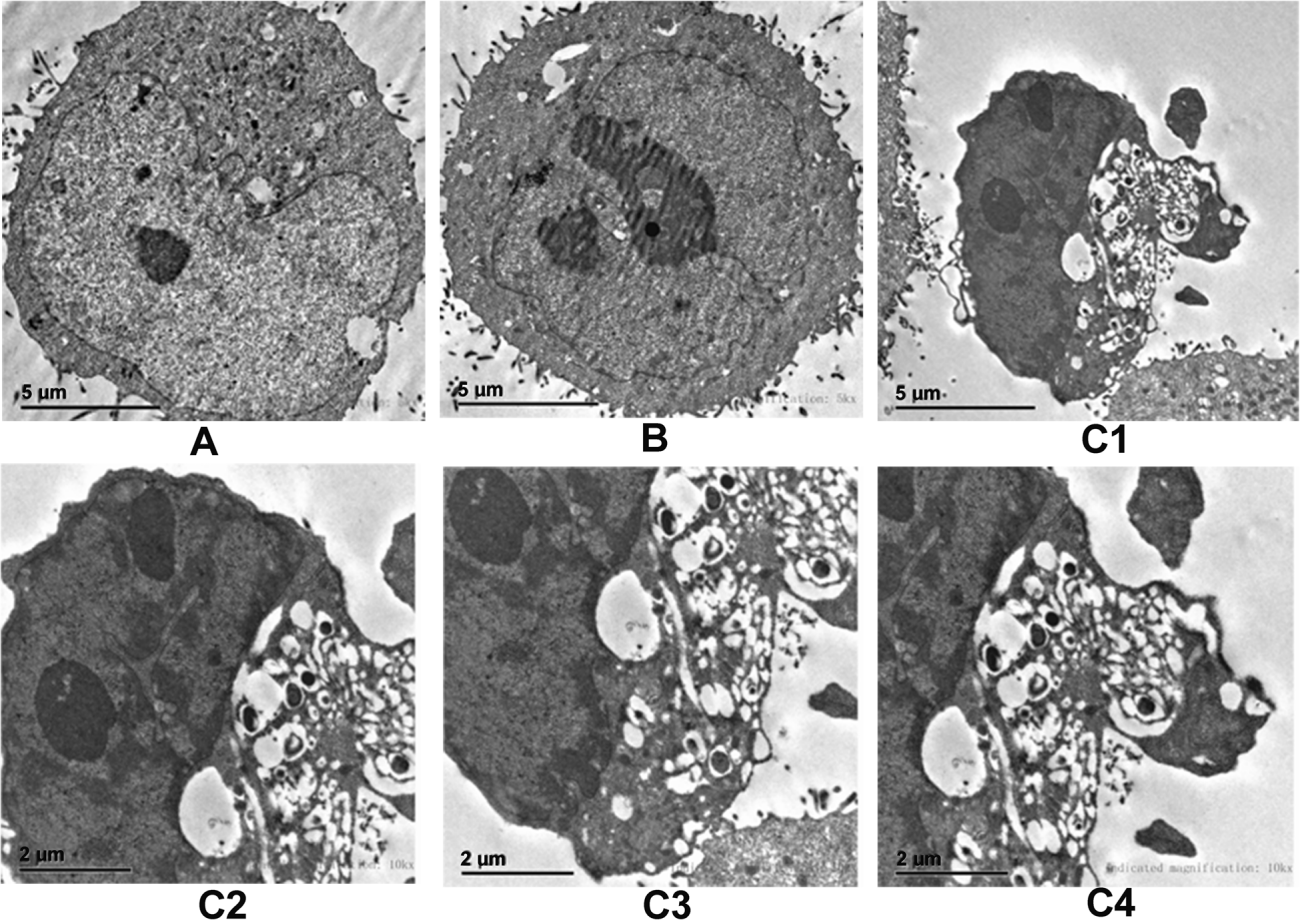
Morphological changes of GES-1 cells as demonstrated by the observed by transmission electron microscopy after incubation at a high final concentration (65 μg/mL) of VacA recombinant protein for 24 h. **A** The blank control (Magnification × 5000). **B** The buffer control (50 mM acetic acid, pH 2.9, Magnification × 5000). C VacA recombinant protein (at a final concentration of 65 μg/mL); **C1:** The cell volume is reduced and the surface microvilli disappears (Magnification × 5000); **C2:** Nucleus shrinks and agglutinates to the periphery (Magnification × 10,000); and **C3 & C4:** Cytoplasmic disintegration and vacuoles (Magnification × 10,000)

**Fig. 7:**
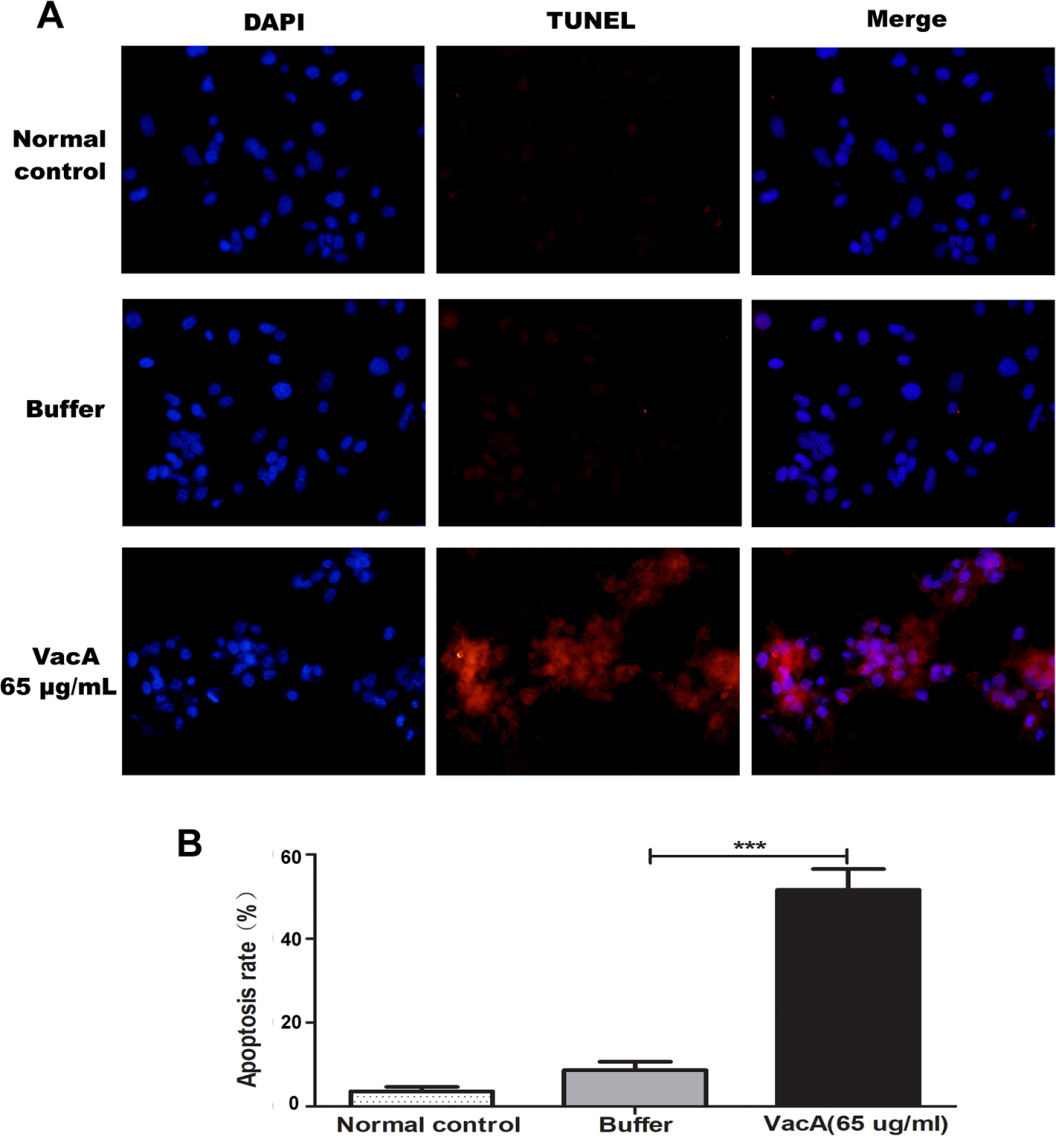
Detection of cells apoptosis induced by VacA recombinant protein using TUNEL assay. VacA recombinant protein induced GES-1 cell apoptosis. GES-1 cells treated with VacA recombinant protein at a high concentration (at a final 65 μg/mL) for 24 h. **A** Representative images of the TUNEL assay (Magnification × 400). Nuclei were counterstained with DAPI (blue) and TUNEL-positive cells (red) indicate apoptosis. **B** Quantification of results. Results are presented as the mean ± standard deviation, ***: *P* < 0.001 vs. the buffer control group

Significant apoptotic activity was observed at a final concentration (65 μg/mL) for 24 h. Under invert microscope and transmission electron microscope, significant cell morphological changes evident of apoptosis including early nuclear immobilization and clustering in GES-1 cells were observed. A large number of nuclei were retracted and chromatin in the nucleus converged to the edge, and the apoptotic activity appeared to be time-dependent (Figs. 5 & 6). In addition, a significant increase of apoptosis was observed as compared to the buffer control group using TUNEL (Fig. 7, *P* < 0.01) assays. No obvious cell vacuolation was observed with the two concentrations at any of the time points (Figs. 5 & 6).

## Discussion

In the present study, a VacA recombinant protein was successfully produced by a recombinant plasmid (*pET41b-VacA*^*34–854*^) in *E. coli* BL21(DE3). In addition, the purified VacA recombinant protein in the inclusion bodies was preserved stably in acetate buffer (pH 2.9) with the biological activity of promoting apoptosis. Therefore, this recombinant VacA can be used as *VacA* toxin to directly perform *H. pylori*-related basic research, providing a tool for further study of *H. pylori* virulence factor *VacA* pathogenic mechanism.

In contrast to the endogenous expression of the *vacA* toxin gene in cells, we chose to construct the VacA recombinant protein for subsequent basic experiments. In a previous study, our team screened the *vacAs1m1 H. pylori* clinical strain, a highly virulent strain that is associated with atrophic gastritis, as demonstrated by animal infection models [20]. To further explore the mechanism of *VacA* toxin in *H. pylori*-related gastric precancerous lesions, a *VacA* eukaryotic expression plasmid was constructed and transfected into GES-1 cells (human gastric mucosal epithelial immortalized cells). We found that apoptosis and cell vacuolation were not obvious in this model. The main reason for this phenomenon is that there is a contact effect between *H. pylori* and epithelial cells in the host, and, thus, the endogenous expression of the *vacA* gene in the cell, induced by plasmid transfection technology, does not fully reflect the toxin effect of *VacA*. de Bernard et al. [21] reported that both microinjection of *VacA* in HeLa cells and transfection with plasmids encoding *VacA* in HeLa cells caused the formation of vacuoles. A recent study [22] showed that *VacA* is the only exocrine toxin that is secreted from *H. pylori*, and plays a crucial role through the manipulation of the type II secretion system in target cells. RPTPα/RPTPβ and LRP1 have been identified as cell-surface VacA-binding receptors. These receptors contribute to the internalization of *VacA* into epithelial cells, as well as activating signal transduction pathways, cell death and gastric ulceration. The interaction between *VacA* and target cells is the crucial event in the induction of the toxicity. Hence, the application of ectogenic *VacA* toxin on target cells would provide an improved simulation of the toxic effects of *H. pylori* on epithelial cells in the physiological state.

We successfully obtained a VacA recombinant proteins from the pET41b-VacA^34–854^-*E. coli* BL21(DE3) expression system. Three sources of the *VacA* toxin have been reported in recent studies: 1) the concentrates of supernatants from *H. pylori* liquid culture was used as rough *VacA* [23, 24], 2) filtration and purification of the natural VacA protein [25] 3) production of a VacA recombinant protein [26, 27]. Each of these sources has advantages and disadvantages. The crude *H. pylori* supernatant concentrate has the ability to maintain the intrinsic antigenicity and biological activity of the VacA protein and the method is relatively simple. However, it is difficult to explain and analyze the function of the protein because of the complicated composition. The procedure of filtration and purification of native the VacA protein is complex. Hence, much effort needs to be taken, including culturing massive *H. pylori* cells and collecting the bacteria or supernatant. After the multi-step purification, only a small amount of the target protein is obtained. For production of a VacA recombinant protein, the full-length or fragment of *VacA* genes are amplified and the genetic transformation technique is used to construct an expression vector carrying the target genes. Then, the target genes of the *VacA* toxin are transformed into *E.coli* and the directional expression of *E.coli* is induced to secrete the VacA protein. Preparation of the VacA recombinant protein is relatively simple and large amount of protein can be produced, which may be sufficient for studying the pathogenic mechanism of *H. pylori*. In addition, the available data indicate that the antigenic region and antigenic property of the recombinant VacA protein are similar to those of the native *VacA* antigen, which can be further used for the development of *H. pylori* vaccine and diagnostic kits [28].

In our study, mature fragments of the VacA protein from *H. pylori* strain were amplified, yielding an 89.7-kDa VacA recombinant protein, VacA^34–854^. The *vacA* gene encodes a protoxin, approximately 140 kDa in mass. Both the amino-terminal and carboxy-terminal signal sequence are proteolytically cleaved during the process of *VacA* secretion, and a ~ 90 kDa mature toxin is exported [29]. Mature toxin molecules are released into the extracellular space and the amino-terminal signal sequence is firstly cleaved. Then, a 88 kDa secreted toxin can be further hydrolyzed and processed into two different domains, which have different functions [29]: an N-terminal fragment of 33 kDa (p99, residues 33–311, toxin activity subunit) and a C-terminal fragment of 55 kDa (p55, residues 312–821, binding subunit) [30]. Chang et al. [31] amplified the full-length *VacA* genes in order to construct a *VacA* protoxin. They found that the recombinant *VacA* toxin lost the epithelial apoptosis promoting activity. This indicates that the spatial configuration of the macromolecular protein is influenced by the expression of full- length *VacA* genes in *E.coli*. Therefore, the intrinsic bioactivity of the native toxin could not be seen. Almost all *H. pylori* strains show allelic variations in the three regions of the *vacA* gene: s region, i region and m region [32]. The combination of two different alleles in each region induces the intensity of vacuolar-like changes in gastric epithelial cells [33] and the *H. pylori* strain that produces the VacA protein with a molecular weight of 88 KDa is more virulent. In the present study, we analyzed nucleotide sequences of multiple variants of *vacA* gene that are published in GenBank and found that 5′ terminal sequences show a high homology. Hence, the VacA mature stable protein fragment from *H. pylori* standard strain J99 was amplified, and the VacA recombinant protein with molecular weight of 89.7 kDa was successfully obtained.

In the present study, a larger amount of VacA recombinant protein was purified from inclusion bodies than the supernatant in *E.coli* and this protein had a pro-apoptotic biological activity. Previously, several studies have reported on the preparation of the VacA recombinant protein; however, there is no consensus on the bioactivity of the recombinant VacA protein. Cho et al. [34] applied a DNA amplification technique to construct recombinant the VacA protein p58 fragment, namely VacA^418–799^. A recombinant protein, VacA ^418–799^, with a molecular mass of 45 kDa was purified from the inclusion bodies. This protein inhibited cell growth in gastric carcinoma cells and induced apoptosis in a dose-dependent and cyclin-dependent manner. Manetti et al. [35] used gene transformation technology to induce the expression of the *VacA* toxin secreted from in *E.coli*. Although the 95-kDa VacA polypeptide was abundantly expressed, it completely lacked any biological activity. Gonzalez-Rivera et al. [17] found that both the p33 domain and p55 domain (residues 346 and 347) were required for assembly of *VacA* in the oligomeric structures, and that proteins lacking these sequences fail to cause cell vacuolation. Junaid et al. [16] constructed mature fragment of the VacA protein and showed that the *VacA* toxin was able to induce apoptosis in both human colonic adenocarcinoma cells and Madin-Darby canine kidney epithelial cells. We were able to purify the *VacA* toxin from the supernatant and inclusion bodies, with a larger amount of target protein being obtained from the inclusion bodies than from the supernatant. Generally, when many exogenous proteins are expressed in *E.coli*, they cannot spontaneously fold to produce proteins with certain spatial structures and specific biological functions. Hence, they exist intracellularly, lack biological activities, and are not able to be applied directly. If the inclusion bodies are renaturated, protein folding will recover the biological activities [36, 37]. There are various reports on the recovery and preservation of the VacA recombinant protein. In the present study, several methods that have been reported in previous studies were attempted [15, 17, 38, 39] to induce VacA protein renaturation. However, none of these techniques were able to preserve the proteins stably. Successful protein renaturing was observed following incubation in buffer 6 (20 mM Tris, 2 M urea, 400 mM Arg, 2.5 mM cysteamine, 0.25 mM cystamine, pH 8.5). However, this buffer contained Arg, which has a promoting effect on apoptosis [40]. Thus, the Arg in buffer 6 would interfere with the detection of the pro-apoptosis biological activity of the VacA protein, and its use in the preservation of VacA protein should be avoided if the protein is to be tested for its biological activities. It has been demonstrated that acidic solution is beneficial for the colonization of *H. pylori*, as well as the activation and maintenance of *VacA* toxin biological activities [18]. The specific activities of the core position of *VacA* are triggered following exposure to low pH, which significantly maintains the spatial conformation of the VacA protein [19, 41]. Thus, we finally chose a two-step dialysis method: i.e. the protein was successfully dissolved in buffer 6, and then dialyzed into buffer 7 (50 mM acetic acid, pH 2.9). In the present study, the VacA recombinant protein was successfully renatured, dissolved in buffer 6 and stably stored in buffer 7. This protocol is in accordance with the gastric pathogenic conditions in the physiological status of *H. pylori* [42]. It should be mentioned that the traditional process of acidic activation of recombinant proteins at low pH [18] was eliminated in the present study.

At present, many studies on the three-dimensional structure of *VacA*, especially the p33 and p55 subunits of *VacA*, are based on the analysis of VacA recombinant proteins. K Zhang et al. raised the determination of the 2.4 Å crystal structure of the p55 domain reveals a predominant right-handed β-helix, which could be major milestones in understanding the *VacA* structure [43]. The 19-Å cryoelectron microscopy (cryo-EM) structure of a *VacA* dodecamer further maps the p33 domain to the central core and the p55 domain to the peripheral arms of the flower-like structure [44]. Recent publications reported the high-resolution structure of full-length soluble VacA protein in protomeric and oligomeric forms [45, 46]. It is revealed by the cryo-EM analysis that the models of the 88-kDa full-length VacA protomer are highly conserved among different oligomers and vision a continuous right-handed β-helix made up of two domains with extensive domain-domain interactions. VacA oligomerization plays a vital role in forming ion channels by inserting them into the lipid membrane bilayer. The 88 kDa VacA monomers secreted by VacA are able to assemble into an assortment of water-soluble oligomeric structures, including hexamers, heptamers, dodecamers, and tetradecamers. A recent study suggests that the water-soluble VacA oligomer first dissociates into monomers at a low pH [47]. This is then followed by the binding of *VacA* monomers to the host cell membrane, insertion into the lipid bilayer, oligomerization, and membrane channel formation. However, there still remain many questions to be answered about structure-function relations within the *VacA* toxin and its mechanism of action. In particular, a high-resolution 3D structure of oligomeric *VacA* has yet to be determined, and whether both the p33 and p55 subunits are required for pore-formation at the inner mitochondrial membrane. Our study provides a powerful tool for finding answers about the structure-function relations within the *VacA*. At the same time, the three-dimensional structure can also be analyzed to define whether the mechanisms of action of recombinant *VacA* are similar to those of the native protein.

The VacA recombinant protein obtained by different methods usually has differences in concentration, purity and protein preservation solution. Therefore, the specific amount of the VacA protein used in the experimental study is not uniform. In our study, we started with concentration 5μg/ml, gradually increased the protein concentration, incubated VacA protein with different concentrations and gradients and observed that cell morphological changes at each time point. It was found that apoptosis could be changed after the protein was incubated for a long time with a low concentration or stimulated for a short time with a high concentration, and the effect of high concentration was more significant. Finally, the protein was selected to stimulate cells with a higher concentration of 65μg/ml to test the pro-apoptotic biological activity of the protein and conduct subsequent experiments. The VacA recombinant protein that was preserved in acetic acid buffer was applied to GES-1 cells and was found to promote apoptosis. However, the promotion of vacuolation was not obvious. This is likely due to the influence of several factors, including the *H. pylori* strain, cell strain, incubation time, temperature [31], pH [48] and γ-glutamyltranspeptidase [49]. Thus, further investigation using different *H. pylori* strains, cell lines, incubation times and temperatures is needed.

*VacA* is one of the most important pathogenic factors of *H. pylori* and is attributable to strain-specific variations in toxin activity. It plays an important role in *H. pylori*-related gastric “phlogistic - carcinoma” lesions [9]. The immuno-inflammation effects of *VacA* on epithelial cells are complex, including the promotion of target cell membrane perforation [50], cytoplasmic vacuolation [21], apoptosis induction [34], and the regulation of the immune response by interfering with the signal pathway of T cell proliferation [51]. However, the specific mechanisms of the cytopathy and dysfunction that are caused by *VacA* toxin remain unclear. The ability to obtain a large number of biologically active VacA recombinant proteins will provide a direct tool for the study of *VacA* pathogenesis. Consequently, our study provides a novel method for the preparation, renaturation and stabilization of the VacA recombinant protein and lays a foundation for further exploration into the related disease processes, the pathogenesis of gastric mucosal injury and the occurrence of gastric cancer caused by *H. pylori* virulence factor, *VacA*. This bioactive VacA recombinant protein can be directly applied to gastric epithelial cells to study the dysfunction of “cytoinflammatory injury-proliferation repair” and abnormal immune regulation caused by *H. pylori* infection in further research.

## Conclusion

In this study, an 89.7-kDa VacA recombinant protein was successfully constructed in *E.coli*, purified in large quantities from the inclusion bodies, and stably preserved in acetate buffer, pH 2.9. In addition, the produced VacA recombinant protein has the biological activity of promoting apoptosis.

## Methods

### Cell strain, bacterial strain and plasmids

A human normal gastric epithelial cell line, GES-1, and a *H. pylori* reference strain (J99, ATCC 700824, cagA+/vacA s1m1) were provided by the Cancer Research Institute of Central South University, Changsha, China. *Escherichia coli* strain, TOP10, was purchased from Genscript Biotech (Nanjing) Corp Co., Ltd., Nanjing, China, and *E. coli* BL21(DE3) strain and plasmid *pET41b* were purchased from Novagen Company, Madison, USA.

### Construction of a VacA recombinant expressing vector

Genomic DNA of *H. pylori* was extracted according to the standard protocol for MiniBEST Bacterial Genomic DNA Extraction Kit (Takara Biotechnology Co., Ltd). The *vacA* gene of *H. pylori* (strain J99 / ATCC 700824) was amplified from the sequences (mature protein fragment of *VacA* toxin, expressing amino acids 34 to 858) by polymerase chain reaction (PCR) method using the mutation primers and gene primers (sequence F1: 5’CAATCGTTGGCGGCATCGCTACGGGTACGGCTGTTGGCACGGTTTCGGGCCTGCTTAGTTGGGGACTC3’, sequence F:5’CTTTAAGAAGGAGATATACATATGTTTTTCACCACGGTTATCATTCCGGCAATCGTTGGCGGCATCGCT3’);F1 contained *Nde*I sites, and F contained *Xho*I sites. Conditions were as follows: Pre-denaturation at 96 °C for 5 min; and 25 cycles of 96 °C for 30 s, 57 °C for 30 s, 72 °C for 1 min 20 s and 72 °C for 5 min. The PCR product was digested with *Nde*I and *Xho*I, and inserted into the expression vector plasmid *pET41b* containing C-terminal histidine tag (8His.tag; Novagen Company). The PCR product and the inserted *pET41b* plasmid were then combined at a 10: 1 M ratio in a ligation reaction containing 1× ligation buffer (50 mM Tris-HCl, pH 7.5, 10 mM MgCl2, 1 mM ATP, 1 mM DL-dithiothreitol (DTT), 25% (w/v) polyethylene glycol 8000) with five units of T4 DNA ligase (Gibco BRL, USA) to reach a final volume of 20 μL and incubated overnight at 16 °C. The combination product (10 μL, 300 ng) was mixed with competent cells (100 μL, 1 × 10^8^ cfu/μg, the optical density of the cells at 600 nm reached 0.5–0.8) of *E.coli* TOP10 on ice for 30 min, in heat shock at 42 °C for 60 s, and then instantly incubated in an ice bath for 2 min. The solution was grown in 800 μL Luria-Bertani (LB) medium, preheated to room temperature, on a shaking incubator for 1.5 h at 37 °C. Afterwards, the cells were harvested by centrifugation at 13,523 g for 2 min and removal of 200 μL supernatant. The solution was resuspended with 200 μL LB medium containing 50 μg/mL kanamycin and then the LB medium was evenly spread into a solid LB medium plate. Following this, the plate was reversely placed and cultured at 37 °C overnight. Five well-grown colonies on the solid LB medium plate were selected and inoculated into 5 mL LB medium containing 50 μg/mL kanamycin. Transformants were grown at 37 °C overnight with shaking. Plasmids were extracted by the methods described by Green et al. [52]. Afterwards, the plasmids were digested with *Nde*I and XhoI. The restricted product, a 2502-bp positive clone, was assessed by 1% agarose gel electrophoresis. The correct recombinant clones were then validated by Sanger dideoxy sequencing, with the Applied Biosystems 3730XL DNA Analyzer (Thermo Fisher Scientific, Inc., Waltham, MA, USA).

### Expression and purification of VacA recombinant protein

#### Expression of VacA recombinant protein

Recombinant plasmid containing the *vacA* insert (*pET41b*-vacA^34–854^) was transformed into *E. coli* BL21(DE3) (Novagen Company). Briefly, *E. coli* cells were incubated in 50 mL LB medium containing 50 μg/mL kanamycin, and incubated overnight at 37 °C with shaking at 225 g. Then, the 10-mL pre-culture above was seeded into 20 × 500 mL Terrific Broth containing 50 μg/mL kanamycin in Erlenmeyer flasks, and incubated at 37 °C with shaking at 225 g. When the OD600 value of the culture reached 1.2, IPTG was introduced at the final concentration of 0.5 mM into the culture to induce the protein expression at 15 °C for 16 h with shaking at 225 g. The cell pellet was harvested at 8000 g, maintained at 4 °C for 20 min. Following this, sodium dodecyl sulfate polyacrylamide gel electrophoresis (SDS-PAGE) and western blotting analysis were used to detect the VacA protein in *E.coli* expression in construct p*ET41b.*

#### Purification of VacA recombinant protein

The cell pellet was harvested at 8000 g, at 4 °C for 20 min, and re-suspended with the lysis buffer (50 mM Tris-HCl, 150 mM NaCl, pH 8.0). The cells were lysed using a sonicator (protocol: 3 s on and 6 s off cycles for a total of 15 min at 500 w) and the cell lysate was centrifuged at 14,650 g, at 4 °C for 30 min. The supernatant and inclusion bodies were separately collected for the purification of the VacA recombinant protein as described below.

#### Purification of VacA recombinant protein from the supernatant

The target protein was purified from the supernatant with Ni-IDA (Ni-IDA resin, Genscript Biotech (Nanjing) Corp Co., Ltd). The above-mentioned lysis buffer (50 mM Tris-HCl, 150 mM NaCl, pH 8.0) was used as the column equilibration buffer. The target protein was eluted with a stepwise gradient of imidazole (50 mM Tris-HCl, 150 mM NaCl, pH 8.0 + a gradient concentration of imidazole, i.e. 20, 50, 100 and 500 mM) and then washed with washing buffer (50 mM Tris, 150 mM NaCl, 1% TritonX-114, pH 8.0. The 1% TritonX-114 acts to remove the endotoxin). Western blotting and SDS-PAGE were used to analyze the purification process. According to the results of Western blotting and SDS-PAGE, the target proteins in the lane with the most protein expression were pooled and dialyzed into buffer 1 × PBS, pH 7.4. The dialysis was performed in a 14 kDa cut-off dialysis membrane (VISKASE® Companies, Inc.) for 4 h and the above buffer was replaced with the same fresh buffer (1 × PBS, pH 7.4) for an additional 16 h. After dialysis, the sample was centrifuged at 14,650 g for 30 min and filtered through a 0.22 μm filter (Merck Millipore). The final quality control (QC) included SDS-PAGE along with western blotting.

#### Purification of VacA recombinant protein from inclusion body

For the purification of VacA recombinant protein in the inclusion bodies, the inclusion body pellet was solubilized in the denature buffer (50 mM Tris-HCl, 8 M Urea, pH 8.0) by sonication. The cell precipitate was centrifuged at 14,650 g for 30 min at 4 °C, and the supernatant was used for further purification. Ni-NTA (Profinity IMAC Ni-Charged Resin, Bio-Rad Laboratories, Inc.) affinity chromatography was applied to collect the recombinant protein in the supernatant. The above-mentioned denature buffer was used as the column equilibration buffer, and the target protein was eluted with a stepwise gradient of imidazole (50 mM Tris-HCl, 8 M urea, pH 8.0 + a gradient concentration imidazole, i.e. 20, 500 mM) after being washed with washing buffer (50 mM Tris-HCl, 1% Triton-X114, 8 M urea, 150 mM NaCl, PH 8.0). SDS-PAGE was used to analyze the fractions in the purification process.

### Refolding, preservation and identification of VacA recombinant protein

#### Refolding and preservation of VacA recombinant protein

The target protein eluted in the above inclusion body purification was pooled, and a small-scale refolding test was performed using the following refolding buffers and methods: buffer 1: 50 mM Tris-HCL, 10% Gly, 150 mM NaCl, pH 8; buffer 2: 50 mM Tris-HCL, 10% Gly, 150 mM NaCl, 0.1 mM DTT, pH 8.0; buffer 3: 1 × PBS pH 7.4; buffer 4: 1 × PBS, 10% GLy, 500 mM NaCl, pH 7.4; buffer 5: 1 × PBS, 10% GLy, 500 mM NaCl, 0.1 mM DTT, pH 7.4; buffer 6: 20 mM Tris-HCL, 2 M urea, 400 mM Arg, 2.5 mM cysteamine, 0.25 mM cystamine, pH 8.5; buffer 7: 50 mM acetic acid, pH 2.9. Analysis of protein refolding was based on the protein solubility (when the protein was dissolved in the buffer, the appearance of the visible precipitate indicated refolding failure, whereas the complete dissolution indicated successful refolding), or the results of SDS-PAGE and western blotting (the lysate was collected and the protein content was further analyzed by SDS-PAGE and western blotting). The dialysis was performed in 14 kDa cut-off dialysis membrane for 4 h and for additional 16 h after replacement of the fresh buffer (i.e. the corresponding dialysis buffer in the dialysis method). Then, dialysis was performed in the final buffer (i.e. the corresponding dialysis buffer in the dialysis method) for 16 h. The most suitable protein final solution was selected based on protein refolding results as well as the buffer solvent used in the solution, which must not interfere with the biological activity detection of the target protein.

#### Identification of VacA recombinant protein

After dialysis, the sample was centrifuged at 14,650 g for 30 min and filtered through a 0.22 μm filter. The final QC included SDS-PAGE along with western blotting.

##### SDS-PAGE analysis

The protein sample was added to the loading buffer (300 mM Tris-HCl, 10% SDS, 30% glycerol, 0.05% bromophenol blue, 250 mM DTT, pH 6.8). The mixture was vortexed for 1 min, heated at 100 °C for 10 min, and centrifuged at 11,270 g for 1 min. The supernatant was taken for SDS-PAGE analysis (Gel: 4% ~ 20% gradient SDS-PAGE gel, Genscript Biotech (Nanjing) Corp Co., Ltd). Equal amounts (2 μg) of bovine serum albumin, (BSA, 67 kDa) and the protein were added to the loading buffer. The next steps were performed as described above. After the electrophoresis was completed, the band of the target protein was compared with the band of the reference protein (BSA), and the amount of the target protein was initially obtained. PAGE-MASTER Protein Standard (Genscript Biotech (Nanjing) Corp Co., Ltd) was used as a protein molecular weight marker.

##### Western blotting analysis

After SDS-PAGE, the precast gel (4% ~ 20% gradient SDS-PAGE gel, Genscript Biotech (Nanjing) Corp Co., Ltd) was fixed on electro phoretic apparatus and the inside of the electrophoresis tank was topped up with MOPS electrophoresis buffer. Then, the protein samples were added into the gel holes. Easy Western Protein Standard (Genscript Biotech (Nanjing) Corp Co., Ltd) was used as a protein molecular weight marker. SDS-PAGE was run at 140 V for 60 min, and stopped when the bromophenol blue reached the bottom of the separation gel. The gel was removed, and transferred onto the PVDF membrane (Bio-Rad Laboratories, Inc.) by using e-blot (a highly efficient wet protein transfer system). The membrane was washed with 1 × PBST (1× phosphate buffered saline (PBS), 0.06% Tween-20, pH 7.4) buffer twice, for 5 min each time, removed and sealed with rapid sealing liquid (Genscript Biotech (Nanjing) Corp Co., Ltd) for 8 min. The membrane was washed with 1 × PBST buffer twice, for 5 min each time, and then incubated at 4 °C overnight with skin milk containing the primary antibody (anti-His antibody, 4000:1). Then, the membrane was washed again, as described above, and incubated with skin milk containing the second antibody (anti-mouse antibody, 5000:1) for 45 min. Finally, the membrane was washed, as described above, and exposed to X-ray film. The bands were visualized using ECL Western Blotting Substrates (Promega Biotech Co., Ltd).

### Detection of apoptotic activity of VacA recombinant protein

In a pilot experiment, we started with concentration 5μg/ml, gradually increased the protein concentration, incubated VacA protein with different concentrations and gradients and observed that cell morphological changes at each time point. It was found that apoptosis could be changed after the protein was incubated for a long time (48 h) with a low concentration (5μg/ml) or stimulated for a short time (24 h) with a high concentration (65μg/ml), and the effect of high concentration was more significant. Finally, we used a higher concentration (65μg/ml) for further experiments to determine the apoptotic activity of VacA recombinant protein as described below.

#### The morphological observation

GES-1 cells were cultured in RPMI 1640 medium (HyClone; GE Healthcare Life Sciences) containing 10% fetal bovine serum (Biological Industries Israel Beit-Haemek, Ltd) that was replaced daily, with trypsinization. Well-grown GES-1 cells were seeded into each well of a 6-well plate, containing 5 × 10^6^ cells, and incubated with VacA recombinant protein (with a final concentration of 65 μg/mL in the experimental group) in an incubator at 37 °C in an atmosphere of 5% CO_2_ for 24 h. At the same time, other batches of GES-1 cells were incubated with RPMI 1640 medium and incubated with isovolumetric protein buffers (buffer 7). These cells served as a blank control group and buffer control group. The plate was removed from the incubator at a later point. Cellular structural changes, including nuclear cytoplasmic apoptosis and cytoplasmic vacuolization, were visualized before incubation and after 6, 12 and 24 h of incubation by invert microscopy. After incubation for 24 h, a single cell suspension was prepared by trypsinization and the samples were made into pathological sections according to conventional methods and examined by transmission electron microscopy, as previously described [53].

#### The terminal deoxynucleotidyl transferase- (TdT-) mediated dUTP nick end labeling (TUNEL) assay

Analysis of apoptotic cells was performed using TUNEL assay kit (Beyotime Biotechnology, Shanghai, China) according to the manufacturer’s instructions. GES-1 cells grown on glass coverslips in 6-well plates were treated with VacA recombinant protein (with a final concentration of 65 μg/mL in the experimental group) as described above, after incubation for 24 h, cells were then washed with PBS, fixed with 4% paraformaldehyde in PBS at room temperature for 30 min, and washed once with PBS. Then, cells were permeabilized in 0.5% Triton X-100 for 5 min and washed twice with PBS. The cells in each well were then incubated with 100 μL of TUNEL reaction mixture containing TdT and Cyanine 3-dUTP at 37 °C in the dark for 1 h. After incubation, cells were washed three times with PBS and mounted in antifade mounting medium containing DAPI. The Cyanine 3-labeled TUNEL-positive cells were captured using a fluorescent microscopy at 400× magnification by using 550 nm excitation and 570 nm emission (red fluorescence). The nuclei were counterstained with DAPI, and the TUNEL-positive cells with red fluorescent staining, indicative of apoptosis. Cells from 20 images for each sample were counted and the percent of apoptotic cells was calculated by dividing the number of apoptotic cells by the number of total cells counted. All assays were performed in triplicate.

### Statistical analysis

All the data were expressed as mean ± standard deviation (SD) and the statistical analysis was performed using SPSS software (version 18.0; SPSS, Inc., Chicago, IL, USA). One-way analysis of variance (ANOVA), with Tukey-Kramer multiple comparison procedure, was used to determine the significance of differences among groups, Statistical significance was set at *P* < 0.05 .

## Supplementary Information

**Additional file 1: Supplementary Figure 1.** Nucleotide and deduced putative amino acid sequences of recombinant *vacA* toxin gene from *H. pylori* strain ATCC 700824.

## Data Availability

The sequence data generated and analyzed in this study are available at Genbank (https://www.ncbi.nlm.nih.gov/genbank) under accession numbers (BankIt2469506 C0144BE060–1 MZ369157). All data generated or analysed during this study are included in this published article.

## Abbreviations

*VacA*: Vacuolating cytotoxin A
TUNEL: The terminal deoxynucleotidyl transferase- (TdT-) mediated dUTP nick end labeling.
