# Supplementary material for: Construction and preservation of a stable and highly expressed recombinant Helicobacter pylori vacuolating cytotoxin A with apoptotic activity
Source: BMC Microbiol. 2021 Aug 18;21:229. doi: 10.1186/s12866-021-02262-7 (PMC8371779; doi:10.1186/s12866-021-02262-7)
Supplement: Supplementary file 1 — Additional file 1: Supplementary Figure 1. Nucleotide and deduced putative amino acid sequences of recombinant vacA toxin gene from H. pylori strain ATCC 700824. [file 12866_2021_2262_MOESM1_ESM.docx]

**Supplementary Figure 1.** Nucleotide and deduced putative amino acid sequences of recombinant *vacA* toxin gene from *H. pylori* strain ATCC 700824.

Note: Underlined areas indicate the position of primers (*vacA* (34-854) + 8His. tag), with a gene length of 2502 bp and a peptide length of 829 aa.

1 CATATGTTTT TCACCACGGT TATCATTCCG GCAATCGTTG GCGGCATCGC TACGGGTACG

1 M F F T T V I I P A I V G G I A T G T A

61 GCTGTTGGCA CGGTTTCGGG CCTGCTTAGT TGGGGACTCA AACAAGCCGA AGAAGCGAAT

21 V G T V S G L L S W G L K Q A E E A N K

121 AAAACCCCAG ATAAACCCGA TAAAGTTTGG CGCATTCAAG CAGGAAAAGG CTTTAATGAA

41 T P D K P D K V W R I Q A G K G F N E F

181 TTCCCTAACA AGGAATACGA CTTATACAAA TCCCTTTTAT CCAGTAAGAT TGATGGAGGT

61 P N K E Y D L Y K S L L S S K I D G G W

241 TGGGACTGGG GGAACGCCGC TAGGCATTAT TGGGTCAAAG GCGGGCAATG GAACAAGCTT

81 D W G N A A R H Y W V K G G Q W N K L E

301 GAAGTGGATA TGAAAGACGC TGTAGGGACT TATAAACTAT CAGGGCTTAG AAACTTTACT

101 V D M K D A V G T Y K L S G L R N F T G

361 GGTGGGGATT TAGACGTGAA TATGCAAAAA GCCACTTTGC GTTTGGGCCA ATTCAATGGC

121 G D L D V N M Q K A T L R L G Q F N G N

421 AATTCTTTCA CAAGCTATAA GGATAGCGCT GATCGCACCA CGAGAGTGAA TTTCAACGCT

141 S F T S Y K D S A D R T T R V N F N A K

481 AAAAATATTT CAATTGATAA TTTTGTAGAA ATCAATAATC GTGTGGGTTC TGGAGCCGGG

161 N I S I D N F V E I N N R V G S G A G R

541 AGAAAAGCCA GCTCTACGGT TTTGACTTTG CAAGCTTCAG AAGGGATCAC TAGCAGTAAA

181 K A S S T V L T L Q A S E G I T S S K N

601 AATGCGGAAA TTTCTCTTTA TGATGGCGCC ACGCTCAATT TGGCTTCAAA CAGCGTTAAA

201 A E I S L Y D G A T L N L A S N S V K L

661 TTAAATGGTA ATGTGTGGAT GGGCCGTTTG CAATACGTGG GAGCGTATTT AGCCCCTTCA

221 N G N V W M G R L Q Y V G A Y L A P S Y

721 TACAGCACGA TCAACACTTC AAAAGTTCAA GGGGAAGTGG ATTTTAACCA TCTCACTGTG

241 S T I N T S K V Q G E V D F N H L T V G

781 GGGGATCAAA ACGCCGCTCA AGCGGGCATT ATCGCTAGCA ATAAGACTCA TATTGGCACA

261 D Q N A A Q A G I I A S N K T H I G T L

841 CTGGATTTGT GGCAAAGCGC CGGGTTAAAT ATCATTGCCC CTCCAGAAGG TGGCTACAAG

281 D L W Q S A G L N I I A P P E G G Y K D

901 GATAAACCTA ATAGTACCAC TTCTCAAAGT GGCACTAAAA ACGACAAGAA AGAGATCAGT

301 K P N S T T S Q S G T K N D K K E I S Q

961 CAAAATAACA ATAGCAACAC AGAGGTCATT AACCCACCCA ATAACACGCA AAAAACAGAA

321 N N N S N T E V I N P P N N T Q K T E T

1021 ACTGAACCCA CGCAAGTCAT TGATGGGCCT TTTGCTGGCG GCAAAGACAC GGTTGTCAAT

341 E P T Q V I D G P F A G G K D T V V N I

1081 ATTTTCCACT TAAACACTAA AGCCGATGGC ACGATTAAAG TGGGAGGGTT TAAAGCTTCT

361 F H L N T K A D G T I K V G G F K A S L

1141 CTTACCACGA ATGCGGCTCA TTTGAATATC GGCAAAGGCG GTGTCAATCT GTCCAATCAA

381 T T N A A H L N I G K G G V N L S N Q A

1201 GCGAGCGGGC GCACCCTTTT AGTGGAAAAT CTAACCGGGA ATATCACCGT TGATGGGCCT

401 S G R T L L V E N L T G N I T V D G P L

1261 TTAAGAGTGA ATAATCAAGT GGGTGGCTAT GCTTTGGCAG GATCAAGCGC GAATTTTGAG

421 R V N N Q V G G Y A L A G S S A N F E F

1321 TTTAAGGCTG GTGTGGATAC TAAAAACGGC ACAGCCACTT TCAATAACGA TATTAGTTTG

441 K A G V D T K N G T A T F N N D I S L G

1381 GGAAGATTTG TGAATTTAAA GGTGGATGCT CATACAGCTA ATTTTAAAGG TATTGATACG

461 R F V N L K V D A H T A N F K G I D T G

1441 GGTAATGGTG GTTTCAACAC CTTAGATTTT AGTGGTGTTA CAGACAAAGT CAATATCAAC

481 N G G F N T L D F S G V T D K V N I N K

1501 AAGCTCATCA CAGCTTCCAC TAATGTGGCC GTTAAAAACT TCAACATTAA TGAATTGATT

501 L I T A S T N V A V K N F N I N E L I V

1561 GTTAAAACCA ATGGGATAAG TGTGGGGGAA TACACTCATT TTAGCGAAGA TATAGGCAGT

521 K T N G I S V G E Y T H F S E D I G S Q

1621 CAATCGCGTA TCAATACCGT GCGTTTGGAA ACTGGCACTA GGTCAATCTT TTCTGGGGGT

541 S R I N T V R L E T G T R S I F S G G V

1681 GTCAAATTTA AAAGCGGTGA AAAACTAGTT ATCAATGATT TTTACTATAG CCCTTGGAAT

561 K F K S G E K L V I N D F Y Y S P W N Y

1741 TATTTTGACG CTAGGAATGT TAAAAATGTT GAAATCACCA GAAAATTCGC TTCTTCAACC

581 F D A R N V K N V E I T R K F A S S T P

1801 CCAGAAAACC CTTGGGGCAC ATCAAAGCTC ATGTTTAATA ATCTAACCTT GGGTCAAAAT

601 E N P W G T S K L M F N N L T L G Q N A

1861 GCGGTCATGG ACTATAGTCA ATTTTCAAAT TTAACCATTC AGGGGGATTT TATCAACAAT

621 V M D Y S Q F S N L T I Q G D F I N N Q

1921 CAAGGCACTA TCAACTATCT GGTCCGAGGC GGGAAAGTGG CAACCTTAAA TGTAGGCAAT

641 G T I N Y L V R G G K V A T L N V G N A

1981 GCAGCAGCTA TGATGTTTAA TAATGATATA GACAGCGCGA CCGGATTTTA CAAACCGCTC

661 A A M M F N N D I D S A T G F Y K P L I

2041 ATCAAGATTA ACAGCGCTCA AGATCTCATT AAAAATACAG AGCATGTTTT ATTGAAAGCG

681 K I N S A Q D L I K N T E H V L L K A K

2101 AAAATCATTG GTTATGGTAA TGTTTCTACA GGTACCAATG GCATTAGTAA TGTTAATCTA

701 I I G Y G N V S T G T N G I S N V N L E

2161 GAAGAGCAAT TCAAAGAGCG CCTAGCCCTT TATAACAATA ATAACCGCAT GGATACTTGT

721 E Q F K E R L A L Y N N N N R M D T C V

2221 GTGGTGCGAA ATACTGATGA CATTAAAGCA TGCGGTATGG CTATCGGCAA TCAAAGCATG

741 V R N T D D I K A C G M A I G N Q S M V

2281 GTGAACAACC CTGACAATTA CAAGTATCTT ATCGGTAAGG CATGGAGAAA TATAGGCATC

761 N N P D N Y K Y L I G K A W R N I G I S

2341 AGTAAAACGG CTAACGGCTC TAAAATTTCG GTGTATTATT TAGGCAATTC TACGCCTACT

781 K T A N G S K I S V Y Y L G N S T P T E

2401 GAGAATGGTG GCAATACCAC CAACTTACCC ACAAACACCA CTAATAATGC GCATTCTGCT

801 N G G N T T N L P T N T T N N A H S A N

2461 AACTACCATC ATCACCACCA TCACCACCAC TAATGACTCG AG

821 Y H H H H H H H H
